# Moral Association Graph: A Cognitive Model for Automated Moral Inference

**DOI:** 10.1111/tops.12774

**Published:** 2024-11-25

**Authors:** Aida Ramezani, Yang Xu

**Affiliations:** ^1^ Department of Computer Science University of Toronto; ^2^ Cognitive Science Program University of Toronto

**Keywords:** Moral inference, Moralization, Word association, Language model, Artificial intelligence

## Abstract

Automated moral inference is an emerging topic of critical importance in artificial intelligence. The contemporary approach typically relies on language models to infer moral relevance or moral properties of a concept. This approach demands complex parameterization and costly computation, and it tends to disconnect with existing psychological accounts of moralization. We present a simple cognitive model for moral inference, *Moral Association Graph (MAG)*, inspired by psychological work on moralization. Our model builds on word association network for inferring moral relevance and draws on rich psychological data. We demonstrate that MAG performs competitively to state‐of‐the‐art language models when evaluated against a comprehensive set of data for automated inference of moral norms and moral judgment of concepts, and in‐context moral inference. We also show that our model yields interpretable outputs and is applicable to informing short‐term moral change.

## Abstract 2

Moral Association Graph (MAG) is a cognitively grounded model for automated moral inference. MAG draws on the psychological data of word association and makes predictionsNot about people's moral perception and judgment. MAG performs competitively and offers an alternative approach to state‐of‐the‐art language models.

## 1 Introduction

Aligning artificial intelligence (AI) systems with human values is one of the most critical challenges we face today, and one prerequisite to tackling this issue is to understand how human values work. Morality plays a central role in societal and individual values, and recent development in AI has offered new tools for studying human moral values at a comprehensive scale. A core approach to this development is automated moral inference, or machine inference of right from wrong with minimal human intervention, which typically draws on information from large text corpora through language modeling. Are language models the best and only way to do moral inference? Here, we offer a simple alternative approach that grounds moral inference in psychological theories of moralization and human semantic network.

There has been a growing interest in connecting AI with human morality. Development over the past decade includes the collection of large‐scale moral judgments (Forbes, Hwang, Shwartz, Sap, & Choi, 2020; Hendrycks et al., 2021; Hoover et al., 2020; Sap et al., 2020; Trager et al., 2022), machine inference of moral values from text (Emelin, Le Bras, Hwang, Forbes, & Choi, 2021; Garten, Boghrati, Hoover, Johnson, & Dehghani, 2016; Jia & Krettenauer, 2017; Johnson & Goldwasser, 2018; Liscio, Dondera, Geadau, Jonker, & Murukannaiah, 2022; Mooijman, Hoover, Lin, Ji, & Dehghani, 2018; Roy, Pacheco, & Goldwasser, 2021; Trager et al., 2022; Xie, Hirst, & Xu, 2020), machine prediction of moral norms (Haemmerl et al., 2023; Jentzsch, Schramowski, Rothkopf, & Kersting, 2019; Ramezani & Xu, 2023; Schramowski, Turan, Jentzsch, Rothkopf, & Kersting, 2019; Schramowski, Turan, Andersen, Rothkopf, & Kersting, 2022), and alignment of AI systems with human moral judgments (Ammanabrolu, Jiang, Sap, Hajishirzi, & Choi, 2022; Jiang et al., 2021; Liu, Zhang, Feng, & Vosoughi, 2022b; Lourie, Le Bras, & Choi, 2021). These recent advances in moral inference from large text corpora allow us to analyze human moral values at an unprecedented scale, but this line of research is often disengaged with psychological accounts describing how human morality works.

One area of significant relevance coming from moral psychology is moralization, the process in which something that previously had no moral relevance becomes associated with moral values (Rozin, Markwith, & Stoess, 1997; Rozin, 1999). Moralization constantly shapes our moral values toward activities such as smoking cigarettes (Rozin & Singh, 1999) and consuming meat (Feinberg, Kovacheff, Teper, & Inbar, 2019), concepts such as new technologies (e.g., GMOs as in Clifford (2019), Inbar, Phelps, and Rozin (2020)), as well as individuals (e.g., political leaders as in Brandt, Wisneski, & Skitka (2015)). Existing work has identified potential factors that influence people's moralization of concepts. Some factors relate to one's rationalization of perceived harms and benefits. For instance, people were found to moralize “eating meat” by associating that with the act of killing animals (Feinberg et al., 2019). Some factors also depend on emotion including the feeling of disgust, for example, toward cigarettes or smoking (Brandt et al., 2015; Rozin & Singh, 1999; Skitka, Wisneski, & Brandt, 2018). These studies offer valuable insights into the psychological basis of moralization, but they often rely on case studies and an experimental setting which can be limiting. Our goal in this study is to develop new computational models informed by psychological accounts of moralization for scalable and interpretable moral inference.

**Fig. 1:**
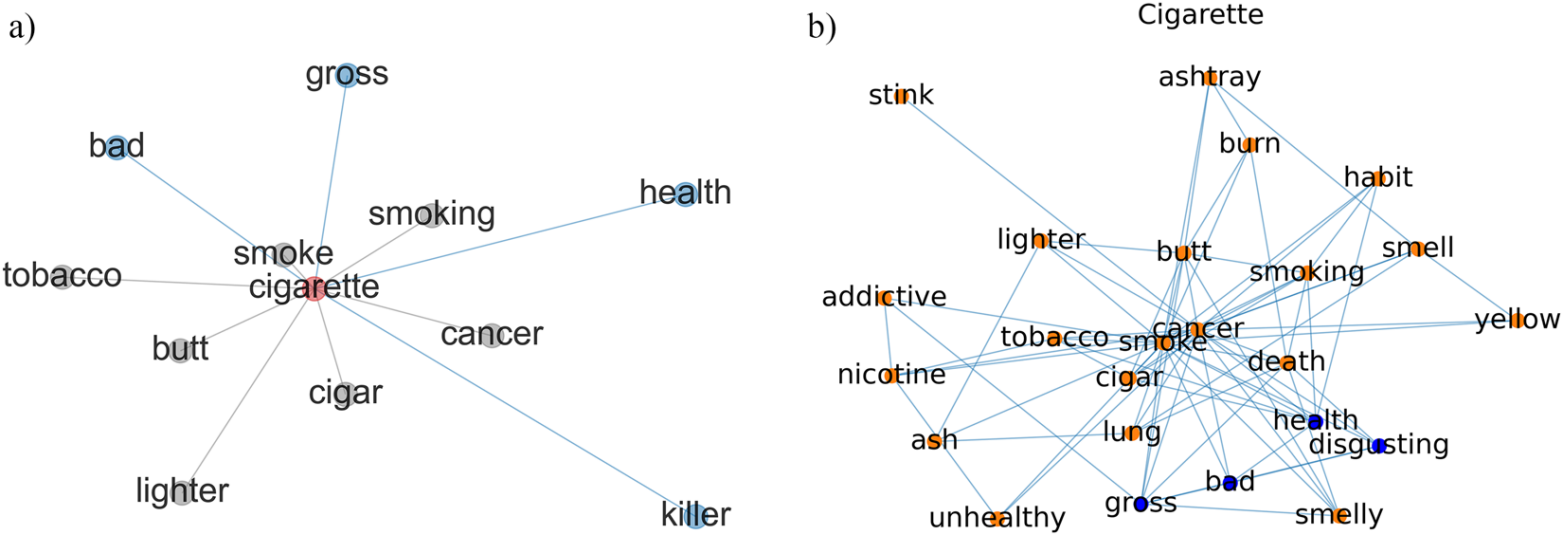
Illustrations of the Moral Association Graph framework based on human word association. (a) A simplified word association network for the example query concept *cigarette*. The edges indicate a directed forward link from the query word *cigarette* to the associated words responded by human participants. Nodes colored in blue indicate morally relevant terms (e.g., *bad, killer, gross*, and *health*). (b) Response network used for interpreting moral inference of the query concept *cigarette*. The edges indicate whether two concepts are consistently associated in response to the query by the same participant, and the blue nodes indicate morally relevant concepts (e.g., *disgusting*, *gross*). Edge weights and directions are omitted for visual clarity in both plots.

We propose the Moral Association Graph model (abbreviated as *MAG*), a computational framework designed to support intuitive moral inference grounded in human word association network (see Fig. 1a). Word association is derived from a psychological game involving participants who are presented with cue words and prompted to respond with the first word(s) coming to their mind (e.g., *cigarette* → *nicotine*). Data collected from word association experiments reflect how words or concepts are mentally represented and connected to one another and serve as a proxy of human semantic network (Collins & Quillian, 1969) and mental representations of word meaning (Deese, 1965; De Deyne et al., 2019; Liu, Cohn, De Deyne, & Frermann, 2022a; Nelson, McEvoy, & Schreiber, 2004; Van Rensbergen, Storms, & De Deyne, 2015). Previous work has also shown that word association better captures human semantics, for example, via representing multimodal properties of concepts, in comparison to distributional semantic language models trained on text (De Deyne, Navarro, Collell, & Perfors, 2021; De Deyne, Cabana, Li, Cai, & McKague, 2020).

We consider word association network as an alternative to language models for moral inference because it captures people's immediate and intuitive reactions, some of which reflect moral perception (e.g., *cigarette* → *gross*). Moreover, our approach is extremely simple: It requires no model training or fine‐tuning, and it is parameter‐free. Despite its simplicity, to our knowledge, formal approach to moral inference with word association is an under‐explored area. We show in a series of analyses that our moral association model either outperforms or achieves comparable performance to state‐of‐the‐art generative language models in inferring people's moral norms and moral judgments at both conceptual and contextual levels. We also demonstrate how our model extracts meaningful key concepts that underlie people's moral judgment, and we apply our model to uncover short‐term changes in moral perception during the COVID‐19 pandemic.

## 2 Computational framework

We describe our computational framework which is inspired by psychological work on moralization and built on psychological data of word association. We also describe the baseline models including large language models.

### 2.1 Moral association graph

This is the main model we propose. The MAG model captures the strength of association between a query (or cue) word and target moral words based on graphs of word association. For example, a cue word like *cigarette* can be associated with concepts such as *bad, death, unhealthy*, which are moral words. We hypothesize that MAG should capture people's moral intuition, particularly how they moralize concepts (Feinberg et al., 2019; Rozin & Singh, 1999). To formulate MAG, we use a dictionary of moral words denoted by M, and the adjacency matrix A. A is an asymmetric matrix of word association, where the rows represent the cue words and the columns represent the response or target words. Each entry, denoted by $A_{c,t}$, represents the number of participants who responded with the target word t given the cue word c ($A_{c,t}$ captures the association strength). MAG is formally specified as follows:

(1) $$MAG\left(c\right)=\frac{\sum_{t\in T(c)\cap M}A_{c,t}}{\sum_{t\in T(c)}A_{c,t}}$$

Here, T(c) is the set of all target responses for the cue word c. This equation measures the proportion of target responses that falls within the moral lexicon, and we take the resulting score as an indicator of moral relevance. To operationalize this model, we draw on the Small World of Words project (SWOW), a large‐scale dataset of word association, which covers more than 12,000 words for English (SWOW‐EN) (De Deyne, Navarro, Perfors, Brysbaert, & Storms, 2019), Dutch (SWOW‐NL) (De Deyne, Navarro, & Storms, 2013), and Rioplatense Spanish (SWOW‐RP) (Cabana, Zugarramurdi, Valle‐Lisboa, & De Deyne, 2023). We gather first‐order associations which offer information about the immediate mental connections among concepts. The responses in SWOW‐EN are predominantly sourced from native English speakers in the United States. Participants in SWOW‐NL are mainly located in Belgium and the Netherlands, and the SWOW‐RP, focused on Rioplatense Spanish which is spoken in Latin America, includes responses from participants in Argentina and Uruguay. The SWOW‐EN and SWOW‐NL cover 100 annotations per cue word, while the SWOW‐RP covers 70. The responses in all the three datasets are normalized and spell‐checked. Additionally, we use the nltk package for lemmatizing the words in SWOW‐EN.^1^

We use Moral Foundations Dictionary (MFD) (Graham, Haidt, & Nosek, 2009) as our base moral lexicon, which is one of the largest lexical resources developed for the Moral Foundations Theory (Graham et al., 2013), and has been widely used to study people's moral values through linguistic data (Garten et al., 2016; Hoover et al., 2020; Mendelsohn, Tsvetkov, & Jurafsky, 2020; Mooijman et al., 2018; Xie, Ferreira Pinto Junior, Hirst, & Xu, 2019). Using the up‐to‐date version of MFD (Frimer, Haidt, Graham, Dehghani, & Boghrati, 2017), we identify a total of 1705 words as our moral lexicon.

Alternative to moral association, we consider a baseline **Emotion Association Graph model (EAG)** given the role of emotion in moralization (Rozin, 1999). We do so by replacing the moral lexicon with an emotion lexicon in Eq. 1. This model captures the strength of emotions associated with a concept. We use the emotion lexicon specified in a prior study (n=626) (Xu, Stellar, & Xu, 2021), compiled from multiple studies on emotion classification (Ekman, 1999; Fehr & Russell, 1984; Johnson‐Laird & Oatley, 1989; Shaver, Schwartz, Kirson, & O'connor, 1987). Since this dataset is only available in English, we use the Google Translate API (googletrans) to translate these words to Dutch and Spanish.

### 2.2 Baselines using language models

We consider several baselines drawing on existing AI studies on moral inference. One study uses the MFD (Graham, Haidt, & Nosek, 2009) and word embeddings to infer moral relevance of individual concepts (Xie et al., 2019). Here, moral relevance for a query is estimated as a probability distribution based on the proximity of that query to the moral word clusters in semantic space. This model also estimates the probability distribution of a concept with respect to different moral foundations. We replicate this work using Word2Vec embeddings (Mikolov, Sutskever, Chen, Corrado, & Dean, 2013) from Google Ngrams, Dutch embeddings of WikiPedia (Tulkens, Emmery, & Daelemans, 2016), and Spanish Billion word Corpus and Embeddings (Cardellino, 2016) for English, Dutch, and Spanish, respectively.

Other studies have used contextual language models for moral inference (Alhassan, Zhang, & Schlegel, 2022; Jentzsch et al., 2019; Schramowski et al., 2022). In one prominent line of work, bert‐based sentence representations (Reimers & Gurevych, 2019) of a set of morally relevant actions (e.g., killing, helping) are used to construct a morally imbued subspace that distinguishes right from wrong (Jentzsch et al., 2019; Schramowski et al., 2022). The moral score of a query is then determined by the similarity between the query and the moral subspace, where values around 0 indicate moral neutral, while values close to +1 or –1 signify high moral relevance. By using multilingual language models such as xlm‐r (Conneau et al., 2020; Reimers & Gurevych, 2020), this model can be extended to identify moral values in different languages (Haemmerl et al., 2023). For our baseline, we employ the same setting in our experiments to explore the moral values of different concepts in English, Dutch, and Spanish. We use xlm‐r‐100langs‐bert‐base‐nli‐mean‐tokens from the sentence‐transformers package to embed the queries.

Finally, we consider generative large language models, such as gpt‐3, that encode people's moral biases and preferences (Dillion, Tandon, Gu, & Gray, 2023; Fischer, Luczak‐Roesch, & Karl, 2023; Ramezani & Xu, 2023; Simmons, 2023). To compare against our MAG model, we probe gpt‐3.5 and gpt‐4 as strong baselines for identifying both the degree of moral relevance and the keywords for explaining people's moral judgments.^2^ Our prompt design is specified in the Appendix.

## 3 Interpreting MAG model

The MAG framework offers a potential way of interpreting the processes of moralization based on shared patterns in people's word association. For example, health‐related concerns and disgust‐related feelings explain why people regard smoking cigarettes as a moral issue (Rozin & Singh, 1999). Similarly, in word association, if a participant's responses to the cue word *cigarette* are terms such as *wrong*, *smell*, and *nausea*, it suggests a negative evaluation, indicating a shared association between the smell of cigarettes and feeling nauseous. Similar patterns across many participants strengthen the probability that these concepts contribute to the formation of negative moral views on *cigarettes*.

To capture this intuition formally, we develop a procedure for quantifying shared associative responses by utilizing the co‐occurrence relationships among the response words. Specifically, for a given cue word c, we construct an undirected weighted graph (denoted by $G_{c}\left(V,E\right)$) with response words as the nodes. The edge weights represent the number of times two response words were mentioned by the same participant. In our previous example, the words *wrong*, *smell*, and *nausea* would all be connected and form a triangle. Using the moral lexicon described, we then start a random walk that initiates from the moral words in this graph and continues the until convergence.

Fig. 1b visualizes this graph and the moral words for *cigarette*. Using a similar random walk process as the previous work in sentiment inference (Hamilton, Clark, Leskovec, & Jurafsky, 2016), we estimate the probability of arriving at a node during the walk based on its proximity to the words in the moral lexicon in Eq. 2:

(2) $$p^{\left(t+1\right)}=\beta \tilde{A}p^{\left(t\right)}+\left(1-\beta \right)m.$$

(3) $$p_{v}^{\left(0\right)}=\frac{\frac{1}{\left|V\right|}+MAG\left(v\right)}{\sum_{u\in V}MAG\left(u\right)+1}.$$

(4) $$m_{v}=Z\left\{\begin{array}{cc} degree(v) & v\;\text{is a moral word}\\ 0 & \text{otherwise}. \end{array}\right.$$

Here, $\tilde{A}$ is the symmetric normalized adjacency matrix representing graph $G_{c}\left(V,E\right)$. As shown in Eq. 3, $p^{\left(0\right)}$ is a vector of size |V|, where each entry corresponds to the MAG score of the respective word in the word association dataset, plus the smoothing factor of $\frac{1}{\left|V\right|}$. This vector controls the walk to propagate from words with the highest MAG scores (i.e., words with salient moral relevance). In Eq. 4, we define m to be a vector of size |V|. For the moral words in V, their entries in m correspond to their weighted degree in $G_{c}\left(V,E\right)$, and for the rest of the words, it is set to be zero. This vector, further normalized by Z to sum up to 1, guides the random walk to remain close to the moral lexicon. Finally, β is a damping parameter that controls the divergence from moral words to longer paths. After convergence, we retrieve the top K words (K=25) with the highest $p^{\left(t\right)}$ scores, which offers an interpretation for the underlying contexts wherein the cue words may be moralized.

## 4 Datasets for model evaluation

We compare and evaluate the models in three main tasks of automated moral inference, drawing on data of human moral norms, and people's moral judgments of individual concepts and in natural context.

### 4.1 Data for moral norm inference

The World Values Survey (WVS) is a publicly available global research survey investigating people's beliefs and values over the globe (Haerpfer et al., 2021; Inglehart et al., 2014a, 2014b). Previous studies have used text‐based methodologies to predict global ratings in the WVS. Their findings suggest that the way people use language provides valuable insights into their beliefs and values (Arora, Kaffee, & Augenstein, 2023; Ramezani & Xu, 2023). Following these studies, we use the participants' aggregate ratings in the ethical section of WVS as the ground truth for assessing people's moral norms.^3^ The ethical section of the WVS explores moral and ethical values by asking people's stances on issues such as *abortion*. To align the population and time course of WVS with the participants and time course of SWOW projects, we use WVS waves 5, 6, and 7 (2005–2022) in countries including the United States (for SWOW‐EN), the Netherlands (for SWOW‐NL, as Belgium is absent in WVS), Argentina, and Uruguay (for SWOW‐RP). We normalize and use the absolute values of WVS responses, where a score of 0 corresponds to a nonmoral issue and a score of 1 corresponds to a highly moral issue. The number of responses to each question varies from 1000 to 6000 participants.

### 4.2 Data for conceptual moral inference

To evaluate moral inference at the concept level, we use the extended Moral Foundations Dictionary (eMFD) (Hopp, Fisher, Cornell, Huskey, & Weber, 2021) which is a dictionary‐based resource for extracting moral foundational content from text. By using human annotations on 2995 news articles, this dataset provides probability scores to a set of 3270 English words, indicating the likelihood of their mention in an article expressing a certain moral foundation.

### 4.3 Data for contextual moral inference

We use three datasets that evaluate moral inference in natural context. The first dataset is Moral Foundations Twitter Corpus (MFTC) (Hoover et al., 2020), which includes more than 30,000 tweets posted in different social discourses of Baltimore protests, US presidential election of 2016, hate speech language, and so on. Each tweet is annotated by at least three annotators with the moral foundational labels. The annotators can annotate a tweet as “nonmoral” indicating that the tweet discusses no moral issues. In our analysis, tweets that receive the “nonmoral” label from all the annotators are considered as nonmoral, and tweets that receive no “nonmoral” label are considered as morally relevant. We retrieve 9759 moral tweets and 2651 nonmoral tweets from MFTC.

The second dataset is Moral Foundations Reddit Corpus (MFRC) (Trager et al., 2022), which includes more than 16,000 Reddit posts from 12 difference subreddits. Similar to MFRC, each post is annotated by at least three human annotators. We use similar procedures to MFTC identifying 3925 moral posts and 3208 nonmoral posts in MFRC.

The third dataset is SOCIAL‐CHEM 101 (Forbes et al., 2020), which was used to train language models for moral norm analysis. The dataset contains 292,000 short text snippets, called the rules‐of‐thumb (RoT), such as “It's kind to sacrifice your well‐being to take care of a sick person.” We identify 75,615 distinct RoTs labeled to be related to morality or ethics, and 190,658 to be nonmoral. We tokenize and lemmatize the text snippets all three datasets with the nltk package for our experiments.

## 5 Results

### 5.1 Evaluation of moral norm inference

**Fig. 2:**
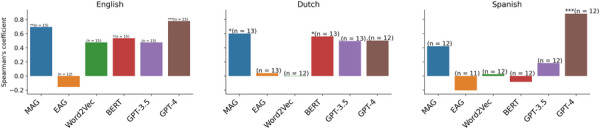
Results of moral norm inference based on correlation between empirical data from World Values Survey and inferred ratings from Moral Association Graph model (MAG), Emotion Association Graph model (EAG), and language model baselines (Word2Vec embedding (Xie et al., 2019), BERT embedding (Haemmerl et al., 2023; Schramowski et al., 2022), gpt‐3.5, and gpt‐4). The asterisks indicate the significance levels (“*,” “**,” “***” for p<.05,.01,.001, respectively) based on Spearman's rank correlation.

We first evaluate models in inferring moral norms across cultures. We use WVS question keywords as queries for our models and intersect those with the word association data we used to construct our models MAG and EAG. We compare the performance of MAG with baseline models including EAG, and language models based on Word2Vec embedding (Xie et al., 2019), BERT embedding (Haemmerl et al., 2023; Schramowski et al., 2022), and gpt. The results in Fig. 2 show that our MAG model consistently outperforms all models (except for gpt‐4) in every case, indicating that the mental associations between different concepts and the moral lexicon capture people's intuitions about the morality of different concepts, without having to be trained on extensive textual data or be refined with human feedback through reinforcement learning (Ouyang et al., 2022). Consistent with previous studies probing morality in language models (Haemmerl et al., 2023), we find that performance of baselines decreases in Dutch and Spanish datasets, suggesting possible misrepresentation of moral values in non‐English language models, and word embeddings. We also note that the MAG model has the lowest performance for Rioplatense Spanish. This dialect is not differentiated from Spanish in Google Translate, which could have hurt the performance of the MAG model, since it uses translated Spanish moral words.

### 5.2 Evaluation of conceptual moral inference

We next assess the models by comparing their predictive outputs for different concepts against the moral foundational probabilities collected in the eMFD (Hopp et al., 2021). In order to evaluate our framework in this fine‐grained setting, we replace the overall MFD dictionary with moral foundational‐specific words and adapt our MAG model to estimate the strength of association to each moral foundation. This modification yields five distinct moral‐foundational association scores for each query concept. We compare these scores with the ground‐truth data in eMFD and summarize the findings in Table 1. From the baselines, only the Word2Vec model developed by Xie et al. (2019) can distinguish between different moral foundations, but we also probe gpt‐3.5 to provide moral foundational scores for different query words. The results show that across all moral foundations, MAG consistently outperforms the Word2Vec and gpt‐3.5 models (Xie et al., 2019), suggesting again that the connections between words and their associative words with moral foundations offer reliable inference at the concept level.

**Table 1:** Results of moral inference at the concept level

Moral Foundation	MAG	Word2Vec	GPT‐3.5
Care/Harm (n=1895)	$0.291^{***}$	0.28	0.28
Fairness/Cheating (n=1514)	$0.232^{***}$	0.193	0.20
Authority/Subversion (n=1737)	$0.301^{***}$	0.192	0.12
Loyalty/Betrayal (n=1714)	$0.212^{***}$	−0.121	0.11
Sanctity/Degradation (n=1893)	$0.246^{***}$	0.222	0.19

*Notes*. The first column shows the moral foundations and sample sizes.

The second and third columns show correlations between empirical eMFD data and our MAG model prediction, Word2Vec and gpt‐3.5 baselines. All values are statistically significant ($p\leq 10^{-4}$) shown with Spearman's rank correlation coefficients.

### 5.3 Evaluation of contextual moral inference

To further assess our framework, we evaluate models on predicting moral relevance in natural sentences. We use the established moral datasets—MFTC (Hoover et al., 2020), MFRC (Trager et al., 2022), and SOCIAL‐CHEM 101 (Forbes et al., 2020) as described. For each text snippet, we lemmatize its words and apply our models to assign a moral score to each word lemma. The overall moral relevance score for an article is calculated based on the average moral scores. Table 2 shows the predictive performance of different models using a correlation test between articles' aggregate moral scores and their moral relevance labels. Similar to the previous experiments, our MAG model reproduces the ground‐truth moral relevance labels outperforming the majority of the baselines and performing on par with the Word2Vec‐based moral sentiment inference model (Xie et al., 2019).

**Table 2:** Results of moral inference in natural context

Model	Dataset 1 MFRC (n=7125)	Dataset 2 MFTC (n=11,910)	Dataset 3 SOCIAL‐CHEM 101 (n=265,898)
MAG	0.475	$0.276^{***}$	$0.244^{***}$
EAG	0.256	0.105	0.067
Word2Vec	$0.522^{***}$	0.266	0.237
BERT	0.329	0.138	0.066
GPT‐3.5	0.308	0.138	0.133
GPT‐4	0.375	0.209	0.187

*Notes*. Different models predict the moral relevance of articles in three datasets: MFRC (Trager et al., 2022), MFTC (Hoover et al., 2020), and SOCIAL‐CHEM 101 (Forbes et al., 2020).

All values are statistically significant ($p\leq 10^{-4}$) shown with coefficients from Spearman's rank correlation.

### 5.4 Retrieving key concepts in moralization

Our MAG model also offers insight into the intuitive process of moralization. Our methodology for keyword retrieval leverages the relationship between first‐level, second‐level, and third‐level association words to uncover the potential cognitive processes that give rise to moralization. Given that there are currently no gold standards for this task, we consider gpt‐4 as a silver standard for measuring the success of our model. For a given query (e.g., *cigarette*), we prompt gpt‐4 to provide a list of keywords explaining the reasons why some people consider this query as a moral issue. Using this silver standard, we assess our framework using the precision, recall, and F1‐score in retrieving the top K=25 keywords suggested by gpt‐4. For the baseline, we use Word2Vec embedding model (Mikolov et al., 2013) trained on the Google Ngrams dataset and identify the top K=25 semantic neighbors of a query term. Using a β value of 0.5, the word association model achieves the precision, recall and F1 values of 0.15, 0.10, and 0.12, while the Word2Vec model achieves 0.04, 0.04, and 0.04, respectively.

In Table 3, we show keywords identified by our association model, gpt‐4, and word2vec for *cigarette*. A quantitative analysis of our framework indicates its capability to identify meaningful connections between moralized concepts and their underlying contexts. For instance, aligned with the theoretical work on the moralization of smoking, our framework identifies keywords such as *cancer, unhealthy*, and *stink*, representing both the rationalization of harms associated with smoking cigarettes and the emotional responses to it (Rozin & Singh, 1999). Although gpt‐4 serves as a competitive baseline for keyword retrieval, we observe that the keywords retrieved by our model exhibit conceptual similarities with those retrieved by gpt‐4. For example, both models retrieve concepts related to *consumerism* and *waste*, offering meaningful insight into the moral reflection of *fashion*, as shown in Table A1.

**Table 3:** Top keywords for the query concept *cigarette*, retrieved from different models for moral inference

Model	Keywords
mag	smoke, health, cancer, unhealthy, tobacco, dirty, cigar, stink, killer, stained, wrong together, smoking, disease, evil, waste, hell, ignorant, death
gpt‐4	health, addiction, secondhand smoke, cancer, harmful, risk, death, pollution, ethical responsibility, choice, disease, cost, danger, unhealthy, lungs, smoke, damage, tobacco habit
word2vec	tobacco, smokes, cigs, cigarrette, marlboros, smoking, ciggie, unfiltered_camels marlboro_lights, newports, pall_malls, winstons, smokeless_tobacco, smokers, menthol_flavored, ciggies, mccargar_bribed, cigarrettes, marlboro_menthol

### 5.5 Applications to quantifying short‐term moral change

**Fig. 3:**
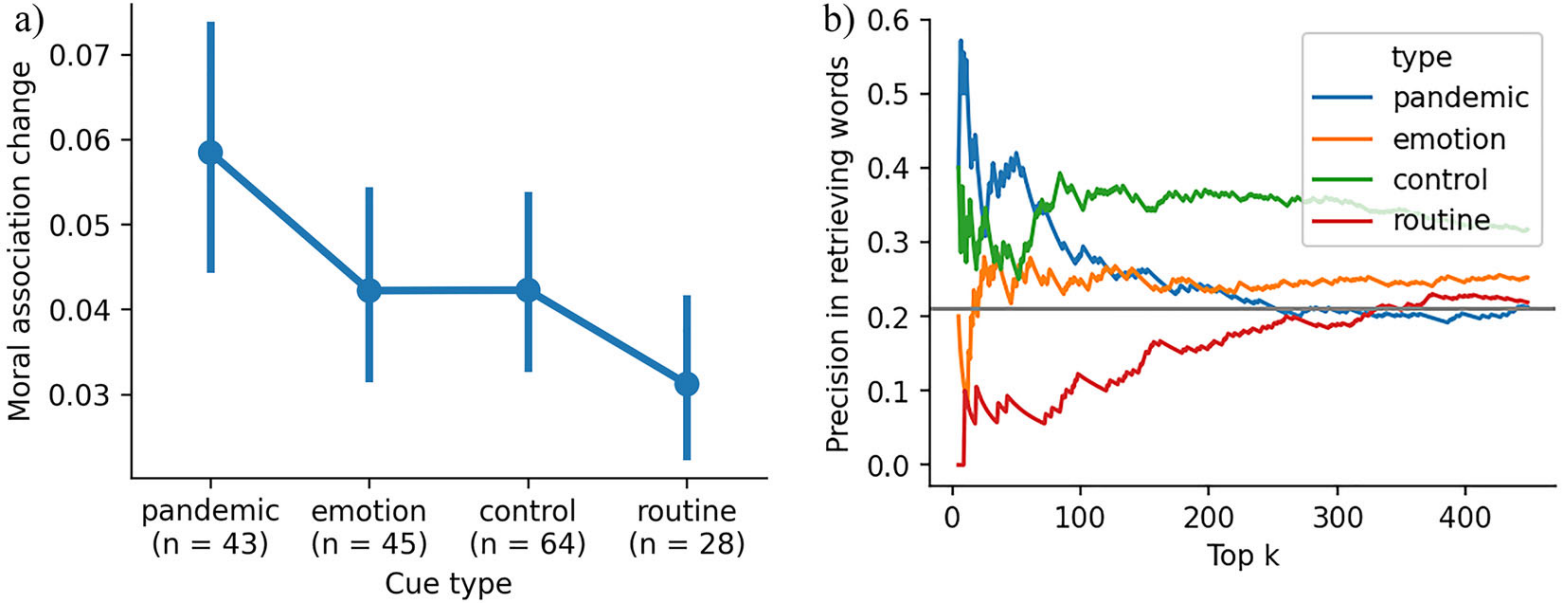
Summary of results on analyzing short‐term moral change in COVID‐19. (a) Degrees of positive change (y‐axis above 0) in moral associations of different word groups. (b) Precision in retrieving top K words with the largest moral association change. The gray horizontal line shows chance level of retrieving the pandemic words.

We apply our framework to identify short‐term changes in people's moral perception in the COVID‐19 pandemic. A section of the SWOW‐RP dataset was gathered post‐December 2020. Within this dataset, a subset of words were collected both before COVID‐19 (December 2013–March 2020) and during the pandemic (December 2020–April 2022). These words were categorized into four groups of pandemic‐related words (*n* = 107), emotion words (*n* = 119), routine words (*n* = 108), and control words (*n* = 150) (Laurino, De Deyne, Cabana, & Kaczer, 2023). Pandemic‐related words refer to those that have gained new meanings or have been excessively used in relation to the pandemic (e.g., *protocol*). Emotion words correspond to feelings that could be affected by the pandemic (e.g., *anxiety*). Routine words describe daily activities impacted by the pandemic (e.g., *tourism*), and control words lack direct connection to the pandemic (e.g., *rain*). Large‐scale experiments on this dataset reveal that pandemic‐related words gained new senses and became more semantically associated with health and sanitary concepts during the pandemic (Laurino et al., 2023). Using the same dataset, we examine whether there are significant changes in the moral association of pandemic‐related words. Specifically, we hypothesize that, among the words that have become more positively associated with moral values, pandemic‐related words should exhibit the most substantial changes. Fig. 3a confirms our hypothesis: we identified 43 pandemic‐related words that have acquired new moral associations. In comparison to the 64 control words with new moral associations, we observed that the degree of moral associations for pandemic‐related words is significantly larger than that for control words (*p*‐value from the Wilcoxon rank‐sum test = .04). Additionally, pandemic‐related words exhibit more substantial changes than routine words (p≤.01). Compared to emotion words, the difference is marginally significant (p=.07). A permutation test comparing pandemic words with other groups confirms a more significant change for pandemic‐related words (p≤.05). Fig. 3b compares the precision in retrieving word groups among the top K words with the highest moral association change. As observed, pandemic terms are the most predominant in the top K words significantly exceeding chance for smaller values of K.

## 6 Conclusion

We present a parameter‐free moral inference model drawing on psychological theories of moralization and data of human word association. In a comprehensive evaluation across half a dozen datasets, we demonstrate that mental association between concepts and moral terms reflects people's moral beliefs about these concepts. Our work offers a promising computational framework for quantifying moral judgment and interpreting the potential mechanisms of moralization. This framework also offers a compelling alternative to language models and helps bridge the gap between the theoretical studies of moralization and computational approaches to automated moral inference. Future research may extend our framework for inferring moralization over extended periods in history and explore its connection with theories of moral reasoning.

## Data and code availability

Data and code for replicating our analyses are available at https://osf.io/pe6qt/?view_only=6781f237174a4eb7ae2b0e826fb2fb8c.

## Notes

1 Access datasets and the metadata at https://smallworldofwords.org/en/project/research.

2 We use the gpt‐3.5‐turbo and gpt‐4 engines and a temperature of 0.5.

3 We follow the terms and conditions for using this resource, detailed at https://www.worldvaluessurvey.org.

## Appendix A

### Prompting of language model

We use the following paragraph to extract moral scores and the underlying factors from gpt‐4. We replace <word> with a cue word, and <number> with 25 in the actual prompt.

Prompt: You will be given a word that represents a particular concept. Provide a number from 0 to 1 that represents the proportion of people in North America who think morally about this concept. For example, if the word is “murder,” the score should be very close to 1, since the majority of people believe “murder” is morally wrong. However, if the given word is “desk,” your score should be close to 0 since “desk” is morally irrelevant. Next, provide a list of words including around <number> cases, separated by commas, explaining why some people would think about this word morally. For example, the explaining words for “murder” could include “wrong, harm, killing, unethical, pain,” and the explaining words for “desk” should be empty. Word: <word>

**Table A1:** Top 25 keywords retrieved from different models for *fashion, environment, abortion*, and *immigration*

Query	Model	Mechanisms
Fashion	mag	waste, whore, slut, clothes, woman, trend, clothing, style, moronic, impractical, appearance consumerism, danger, runway, dress, sense, model, magazine, Paris, accessory design, idiocy, shoulder‐pads, trendy, fashionista
	gpt‐4	exploitation, sweatshops, child labor, animal cruelty, environmental impact, waste, consumerism materialism, body image, vanity, cultural appropriation, fast fashion, unethical production unfair wages, overconsumption, societal pressure, inequality, classism, superficiality, beauty standards sustainability, human rights violations, discrimination, labor rights, pollution.
	word2vec	couture, menswear, ladylike_chic, haute_couture, catwalk, nyfw, fasion, nathan_jenden, emmanuel_ungaro label_edun, men's_wear, luly_yang, prêt_porter, lanvin, flirty_feminine, womenswear, topshop_unique azzedine_alaïa, sportswear, biker_chic, haute_couture, hippy_chic, bianca_spender, lingerie, maxmara
Environment	mag	protect, green, protection, health, nature, world, earth, dirty, safe, trust, law, caring, surroundings, ecology forest, pollution, biosphere, space, planet, area, natural, conservation, film festival, in danger, outside
	gpt‐4	pollution, sustainability, climate change, deforestation, conservation, waste, recycling, responsibility, stewardship biodiversity, eco‐friendly, preservation, global warming, ozone depletion, resource depletion, animal extinction clean energy, carbon footprint, green living, ethical consumption, habitat destruction, overpopulation, water scarcity air quality, renewable resources
	word2vec	evironment, climate, atmosphere, en_vironment, ecosystems, ecosystem, climates, Mike_Taugher_covers conditions, biosphere, landscape, habitats, willyoujoinus.com, regulatory_frameworks, ecology coastal_ecosystems, surroundings, ecosphere, reducing_carbon_footprints, Melissa_McEver_covers threatscape, marketplace, aquatic_biodiversity, aquatic_habitats, trainable_workforce
Abortion	mag	murder, right, baby, death, kill, sad, fetus, legal, ethic, choice, evil, woman, religion, church, help, bad, sin criminal, illegal, good, life, fight, morality, freedom, blood
	gpt‐4	life, right, choice, ethics, morality, baby, fetus, health, religion, belief, controversy, women's rights personal decision, birth control, responsibility, freedom, law, values, safety, dignity, justice autonomy, harm, respect, conscience
	word2vec	abortions, antiabortion, legalized_abortion, pro_lifers, abortionist, abortion_clinics, prolife, legalizing_abortion gay_marriage, prochoice, planned_parenthood, abortionists, opposes_abortion, anti_abortionists terminate_pregnancy, pro_choicers, Roe_v._Wade, contraception, unborn, abortions, antiabortion_activists outlaw_abortions, anti_choicers, pro_abortionists
Immigration	mag	illegal, law, border, legal, Mexican, Mexico, good, emigration, reform, immigrant, injustice, authority, justice foreigner, right, migration, Ellis Island, boat, new, long, misunderstood, people, diversity, visa, refugee, Mexicans emancipation, tax, racism, queue
	gpt‐4	legality, citizenship, economy, jobs, human rights, diversity, culture, security, nationality, asylum, refugees border, policy, law, fairness, equality, opportunity, racism, discrimination, poverty, crime, social cohesion integration, multiculturalism, humanitarian
	word2vec	illegal_immigration, immigrations, illegal_immigrants, immigation, immigrants, illegal_immigrant immigration_reform, illegal_alien, deportations, deportation, immigrant, undocumented_immigrants undocumented_workers, immi_gration, deporting, illegal_aliens, attorney_Ilana_Greenstein deport, illegals, undocumented, undocumented_immigrant, expedited_removal illegal_immigration, undocumented_aliens
